# Asymmetrical Bioimpedance in the Anterior Circulation for Urgent Stratification of suspected Stroke (ABACUS Stroke): study protocol for a diagnostic accuracy study

**DOI:** 10.1186/s41512-019-0068-3

**Published:** 2020-02-20

**Authors:** Christopher I. Price, Lisa Shaw, Anand Dixit, Sara Graziadio, Clare Lendrem, Dipayan Mitra, Helen Rodgers, Lou Sutcliffe, Phil White

**Affiliations:** 1 Stroke Research Group, Population Health Sciences Institute, Newcastle University, Henry Wellcome Building, Framlington Place, Newcastle upon Tyne, NE2 4HH UK; 2 Newcastle upon Tyne Hospitals NHS Foundation Trust, Royal Victoria Hospital, Queen Victoria Road, Newcastle upon Tyne, NE1 4LP UK; 3 NIHR Newcastle In Vitro Diagnostics Co-operative, Room M2.061, William Leech Building, Medical School, Newcastle University, Framlington Place, Newcastle upon Tyne, NE2 4HH UK

**Keywords:** Stroke, Large vessel occlusion, Intracerebral haemorrhage, Cerebrotech Visor™ System, Diagnostic accuracy study

## Abstract

**Background:**

Early identification and treatment of stroke improve outcome. Ischaemic stroke due to large vessel occlusion (LVO) benefits from time-critical thrombectomy but this is only available in highly specialised healthcare services. Cerebral Bioimpedance Asymmetry (CBA) measurement obtained with the portable and rapid Cerebrotech Visor™ System device may be able to identify certain types of stroke including LVO. This test could be deployed pre-hospital and used to immediately direct patients to the most appropriate healthcare service for treatment. This study is evaluating the diagnostic accuracy of CBA measurements obtained from a real-world population of suspected stroke.

**Methods:**

Study design: Prospective observational cohort study.

Setting: A hyperacute stroke unit and neuroscience centre in North East England.

Participants: Adults with a paramedic assigned diagnosis of suspected stroke arriving at hospital within 6 hours of symptom onset.

Index Test: Cerebral Bioimpedance Asymmetry measurement performed using the Cerebrotech Visor™ System. Measurement values produce continuous data (range 0 –100); pre-defined threshold for positive state ≥ 10.

Reference Standard Tests: Standard CT brain +/- CT/MR angiography, and expert clinician opinion will establish the following clinical outcomes which constitute the suspected stroke population: ischaemic stroke +/- large vessel occlusion; symptomatic severe anterior vessel stenosis; large (≥60ml) and small (<60mls) vessel intracerebral haemorrhage; transient ischaemic attack; stroke mimic conditions; prior territorial stroke.

Analyses: Sensitivity, specificity, negative and positive predictive values, area under the Receiver Operating Characteristic curve for identification of i) “complex stroke” (ischaemic stroke with large vessel occlusion or symptomatic severe anterior vessel stenosis or intracerebral haemorrhage ≥60ml or prior territorial stroke) and ii) ischaemic stroke with large vessel occlusion in isolation.

Sample size: 124 participants

**Discussion:**

The results from this study will determine how accurately CBA measurement using the Cerebrotech Visor™ System can identify key stroke types within the suspected stroke population. Acceptable diagnostic performance would be an important step forwards for access to time-critical treatments.

**Trial registration:**

Registered with ISRCTN (identifier: ISRCTN79169844) on 06/08/2018.

## Background

Stroke is responsible for a high global burden of mortality and disability which is likely to increase in line with the ageing population [1]. It is a common medical emergency and in the UK, there are 86,000 admissions per year with an economic impact of £9 billion in health and social care costs [2]. The cause of stroke is either cerebral ischaemia (85%) or haemorrhage (15%) and although all patients benefit from urgent admission to organised multidisciplinary stroke care [3], there is strong evidence that two emergency reperfusion treatments for ischaemic stroke significantly reduce long-term disability: intravenous thrombolysis (up to 15% patients: mean number needed to treat 7) [4, 5] and intra-arterial mechanical thrombectomy (up to 10% patients: mean number needed to treat 3) [6]. The latter is an effective minimally invasive surgical treatment during which an interventional neuro-radiologist extracts thrombus causing large vessel occlusion (LVO).

To achieve optimal benefit, thrombectomy must be performed as soon as possible for patients with moderate-severe clinical stroke severity (National Institutes for Health Stroke Score (NIHSS) [7] > 5 points), and within 6 h of stroke onset according to current clinical guidelines [8, 9], although evidence continues to emerge of later benefit [6, 10]. Without treatment, these patients have a poor outcome because a large clot causes a reduction in blood supply to most of one cerebral hemisphere, and thrombolysis alone is less likely to restore independence [11, 12].

Thrombectomy provision is currently expanding in many countries. In the UK, regional neuroscience ‘hubs’ will provide this treatment for populations of 1.5–3 million. The majority of eligible patients will initially be identified at local Hyperacute Stroke Units (HASU) ‘spokes’ which serve populations of 0.3–1 million. Due to the expertise and facilities required, thrombectomy cannot be provided at every HASU and will only be available in 25 out of 126 sites admitting stroke across England, Wales and Northern Ireland [13]. In this service arrangement, patients presenting at local HASU’s with ischaemic stroke that appear potentially suitable for thombectomy on the basis of clinical stroke severity (NIHSS > 5), radiological criteria and previous medical history will need to undergo additional local CT angiography or MR angiography to confirm the presence of LVO before transfer onto the regional neuroscience hub. This assessment process takes at least one additional hour relative to direct admission to a regional neuroscience hub [14]. In addition, CT/MR angiography is not yet routinely available at all local HASUs in the NHS, and patients admitted to these sites may not be offered thrombectomy treatment as LVO cannot be identified.

Thrombectomy treatment rate and speed would be much improved if patients with LVO stroke could be identified sooner. If this could be done in ambulances, it would enable transportation directly to a regional neuroscience centre. However, reducing the need for additional CT/MR angiography at a local HASU would also be of benefit. In terms of pre-hospital detection of LVO, paramedic completed clinical assessments have been developed to identify LVO stroke patients, but their performance in practice does not support widespread introduction [15].

Whereas there would be global agreement that early recognition of patients with a high probability of LVO could improve thrombectomy outcomes, internationally there are also variations in stroke service provision which impact more broadly upon optimal stratification of patients with stroke. In the UK, there is an expectation that all stroke patients are routinely admitted to a HASU providing specialist multidisciplinary care irrespective of severity or aetiology, and following initial assessment, only a small number are escalated to regional neuroscience centres for specific medical treatments such as thrombectomy. Due to the organisation of healthcare delivery in other nations, there is often greater reliance upon individual clinical need when deciding upon the immediate care destination, i.e. those cases that may require complex assessment and/or treatment are identified for early escalation beyond the first admitting hospital (which may not have a HASU), whereas patients with uncomplicated minor stroke may remain under the most appropriate local speciality. Early identification of this ‘complex’ group could also improve outcomes and resource utilisation in these healthcare systems.

More complex scenarios include symptomatic severe anterior vessel stenosis (SSAVS) of the internal carotid or middle cerebral arteries, large volume intracerebral haemorrhage and patients with a prior territorial stroke (PTS). In SSAVS, although the reduced cerebral perfusion is not due to an occluding thrombus, the clinical presentation of these patients is similar, and they may require advanced neuroimaging and an experienced clinical opinion to determine whether urgent stenting should be offered on a case by case basis [16]. Large volume (≥ 60 ml) intracerebral haemorrhage (LICH) is associated with high morbidity and mortality [17], but early identification could improve outcomes through physiological monitoring, blood pressure control, reversal of coagulopathy, neurosurgical review and advanced imaging to prevent early recurrence [18]. Patients with a prior territorial stroke are at higher risk of further events and early complications, and their immediate management is more likely to require advanced imaging, experienced clinical opinion and decision making, and expert multidisciplinary input.

The recently developed Cerebrotech Visor™ System (CVS) uses the principles of volumetric integral phase-shift spectroscopy to measure and compare alterations in electrical properties of brain tissue (cerebral bioimpedance) resulting from changes in fluids [19, 20]. Low-power electromagnetic waves are transmitted through the brain at multiple frequencies towards a receiver. The phase shifts and attenuations between the transmitter and receiver are sensitive indicators of alterations in brain fluids and can compare fluid changes between cerebral hemispheres providing a measurement of Cerebral Bioimpedance Asymmetry (CBA). The CVS is worn like spectacles over the forehead; it is portable and measurement takes approximately 3 min.

Preliminary data from using the CVS in a hospital setting suggest that most cases of ‘complex stroke’ (defined as ischaemic stroke with LVO, or SSAVS or LICH with ≥ 60 ml haematoma volume, or PTS) can be identified by CBA measurement. A retrospective analysis was able to distinguish between 57 cases of complex stroke and 26 cases of ‘minor stroke’ (defined as ischaemic stroke without LVO or ICH < 60 ml haematoma volume) with 93% (95% CI: 83–98) sensitivity and 92% (95% CI: 75–99) specificity at the optimum CBA threshold [21]. The area under the receiver operating characteristic curve was 93%. Most of the complex stroke cases were ischaemic stroke due to acute LVO (*n* = 41).

Although these preliminary data are encouraging, the patients included were already identified by medical staff as ‘likely stroke’ and as such were not representative of all patients with suspected stroke seen by pre-hospital clinicians or arriving at a HASU. Currently, in the UK, 30–40% of emergency ambulance admissions with suspected stroke are later diagnosed with a stroke ‘mimic’ condition such as seizure or migraine [22]. To be of optimal benefit, the CVS would need to identify patients with complex stroke from within a broad population of suspected stroke.

This manuscript describes the protocol for a study to evaluate the CVS diagnostic performance in a population of unselected suspected stroke arriving at a UK HASU. Whilst the most important future use for the CVS is likely to be in the pre-hospital setting, conducting research evaluations in this context is particularly challenging and prior to embarking on such a study, there needs to be preliminary data of accuracy in a comparable population. This can be achieved by obtaining CBA readings undertaken by trained hospital staff from patients with a paramedic assigned diagnosis of suspected stroke immediately following hospital admission and before specialist or radiological diagnosis is made. This research is timely and topical because finding an accurate, portable and potentially widely available point of care diagnostic for LVO or complex stroke would revolutionise stroke care.

## Methods

### Study objectives

- To determine the diagnostic accuracy of CBA measurement performed using the CVS to identify complex stroke (ischaemic stroke with LVO or SSAVS or LICH ≥ 60 ml in volume or PTS) in patients arriving at a hospital with a paramedic assigned diagnosis of suspected acute stroke.
- To determine the diagnostic accuracy of CBA measurement performed using the CVS to identify LVO in patients arriving at a hospital with a paramedic assigned diagnosis of suspected acute stroke.
- To explore key clinical and radiological influences upon the diagnostic accuracy of CBA measurement for complex stroke and LVO.
- To report the technical failure rate of the CVS.

### Study design

The study design is a prospective blinded observational cohort study. Participants will undergo CBA measurement with the CVS but otherwise will receive standard care and investigations as per current local ambulance and hospital clinical protocols, which are underpinned by national clinical guidelines [8] and audit [13]. The CVS does not display the CBA result which must be generated from raw reading data by the manufacturer. The CBA reading will not be available to participants or any clinical staff involved in routine care. Similarly, clinical data will not be available to the manufacturer prior to the provision of a CBA reading to researchers. There will be no change to participant treatment as a result of the study.

The study will be reported according to the STARD guidelines (Standards for the Reporting of Diagnostic accuracy studies) [23].

### Study setting

The study will take place within a single site (Royal Victoria Infirmary, Newcastle upon Tyne, UK) which receives approximately 1500 direct ambulance admissions of patients with suspected stroke annually. This is a regional neuroscience centre providing thrombectomy within 6 h of symptom onset for patients with an NIHSS > 5 points and LVO proven by CT angiography. Patients will be assessed in the HASU and emergency department.

### Study participants

Trained hospital staff (e.g. research nurses, research trained doctors) will attempt a CBA measurement with the CVS on patients that fulfil the following criteria:

Inclusion criteria:

- Aged 18 years or over
- Transportation to the study hospital by ambulance
- New stroke suspected by ambulance personnel before hospital arrival
- At least responsive to tactile stimuli on hospital arrival—i.e. A, V or P on the Alert Voice Pain Unresponsive (AVPU) scale.
- Routine emergency brain imaging is intended to be performed within 60 min of hospital arrival or has already been performed within 60 min of hospital arrival
- A CBA reading can be attempted +/− 30 min of the first routine emergency brain imaging
- The CBA reading can be attempted within 6 h of a hospital stroke specialist last known well time or symptom onset time.

Exclusion criteria:

- Females who are pregnant, lactating or at risk of pregnancy (i.e. who are not surgically sterile or at least 1 year post last menstrual period). If this information is unknown/cannot be rapidly established, females < 56 years of age will be excluded.
- Already assessed at another hospital and transfer to the regional neurosciences centre is for further investigation or treatment
- Hypoglycaemia (capillary glucose < 3.5 mmol/l)
- Presence of any known implanted electro-stimulating devices in the head and neck
- Presence of implanted hearing aids (removable hearing aids should be taken out before use of the CVS)
- Presence of any known metallic craniofacial implants, such as bone fixation plates (aneurysm coils or clips are acceptable) or cranioplasty
- Recent craniotomy, craniectomy or other reason known for the presence of intracranial air
- Physical inability to wear the investigational device (e.g. skin lesion on scalp, haematoma)
- Any other condition, which in the judgment of the stroke clinician might prevent the patient from tolerating CBA measurement or brain imaging (e.g. severe agitation or requiring immediate intensive treatment unit admission).
- Ambulance staff suspected stroke but hospital staff do not consider that brain imaging within 60 min of hospital arrival is a required investigation (e.g. a different diagnosis is made with other routine assessments or tests shortly after admission)

### Participant identification and consent

Patients will be assessed for study suitability immediately after arrival at the hospital in parallel with their urgent clinical assessment. The regional ambulance service clinical pathway mandates telephone pre-notification to hospital during the conveyance of suspected stroke patients with an onset time of less than 4 h. Upon arrival, the stroke team will rapidly assess these patients including the determinates of eligibility as listed above.

If the patient fulfils the eligibility criteria and staff trained in the use of the CVS are in attendance, a CBA measurement will subsequently be obtained following a short verbal explanation. A formal research consent process will not be undertaken at this point because acute stroke treatments are time-dependent and patients require a rapid assessment to avoid treatment delay. A formal research consent process undertaken before the CBA measurement would cause unacceptable delays. The CBA measurement will be obtained whilst the standard clinical assessment is ongoing.

Once the emergency assessment and treatments are completed, all patients who have had a CBA reading attempted will be approached for study enrolment (including patients where the CVS did not provide data due to technical difficulties or where on review inclusion criteria were not met, e.g. routine brain imaging had been intended but did not take place for some reason).

Patients will be approached about study participation by hospital research trained staff who will either have been involved in CBA measurement or receive direct communication from the clinical team about patients who have undergone a measurement. Ideally, patients will be approached during their inpatient stay such that a timely discussion about the study can be held. However, some stroke and mimic patients are discharged very early after attendance, including directly from the ED. Should a patient who underwent a CBA reading be discharged from the hospital before the consent process can be undertaken, a postal invitation to take part in the study will be used.

#### Consent

The consent process will seek permission for retention and analysis of CBA reading data and collection of selected routinely recorded healthcare data related to acute assessments and treatments (see ‘Study data collection’ section below) which are essential to complete the study objectives. There are no additional study-specific assessments.

#### Consent for patients who can be approached about study participation during their inpatient stay

- Consent for patients with mental capacity. For eligible patients with the capacity to consent to research, a research trained member of staff will approach the patient to discuss the study and provide a patient information sheet. After allowing sufficient time for potential participants to decide whether to take part in the study and an opportunity to ask questions, consent will be obtained in writing.
  When a patient has mental capacity but is unable to sign the consent form (e.g. because of weakness of the dominant hand following stroke), consent will be confirmed orally in the presence of a witness (an individual not otherwise involved in the trial) and the witness will sign and date the consent form on behalf of the participant.
- Consent for patients with mild communication difficulties. For patients with mild communication difficulties due to the effects of stroke or a mimic condition upon the use and understanding of language (aphasia), a set of ‘easy access’ study documentation will be used. After allowing sufficient time for the information to be considered and an opportunity to ask questions, consent will be obtained in writing using the ‘easy access’ consent form.
- Consent for patients who lack mental capacity. It is anticipated that approximately one-third of study eligible patients will be unable to engage with an informed consent process due to the effects of stroke and mimic conditions upon communication and cognition. As exclusion of this group would drastically reduce the clinical relevance of the study, if a patient has been identified as eligible but is unable to provide consent, a personal or nominated (professional) consultee will be approached.
  For potential participants who lack the capacity to consent to research, a trained member of staff will attempt to identify an appropriate personal consultee (usually the next of kin) to approach, discuss the study and provide a consultee information sheet. If a personal consultee is identified, after allowing sufficient time for him/her to consider the patient’s wishes and feelings and an opportunity to ask questions, the consultee will be asked to complete a consultee declaration form if they believe that the patient would have no objection to taking part in the study.
  In the event of being unable to locate an appropriate personal consultee before discharge, an independent clinician (nominated consultee) will be asked to confirm that the patient lacks capacity for consent, and that study participation would not introduce a risk of harm or be against the patient’s wishes from what is known about their character and beliefs. The independent clinician will sign a nominated consultee declaration form concerning study participation.
- Consent and early mortality. The early mortality rate following acute stroke is approximately 10%. Some mimic conditions are also associated with high mortality, e.g. severe infections. However, early unexpected deaths also occur in both stroke and mimic groups. Exclusion of patients who die soon after admission would reduce the study’s relevance for the typical suspected stroke population.
  If a patient who underwent a CBA reading attempt dies before consent can be obtained using one of the approaches described above, the local principal investigator will sign an early mortality declaration form to confirm that the patient has died thus taking responsibility for the use of data collected for this research project.

#### Consent for patients who are discharged from hospital

Patients who are discharged from hospital before the consent process can be undertaken will receive an invitation letter, patient information sheet, consent form and pre-paid return envelope by post. Patients willing to take part in the study will be asked to return a completed consent form. Invited patients who have not returned a consent form within 4 weeks will receive one phone call from the hospital research team to discuss the study.

#### Changes in capacity to consent to research during participation in the study

As there are no additional study-specific assessments and only collection of routinely available healthcare data in this project after the CBA measurement, changes in capacity status will not be reviewed.

#### Consent not obtained

If a patient or a consultee declines the invitation to be included in the study or does not return a postal consent form, or if consent by one of the approaches above is not obtained for any other reason, CBA measurement data will be deleted. No further study data will be collected.

**Fig. 1:**
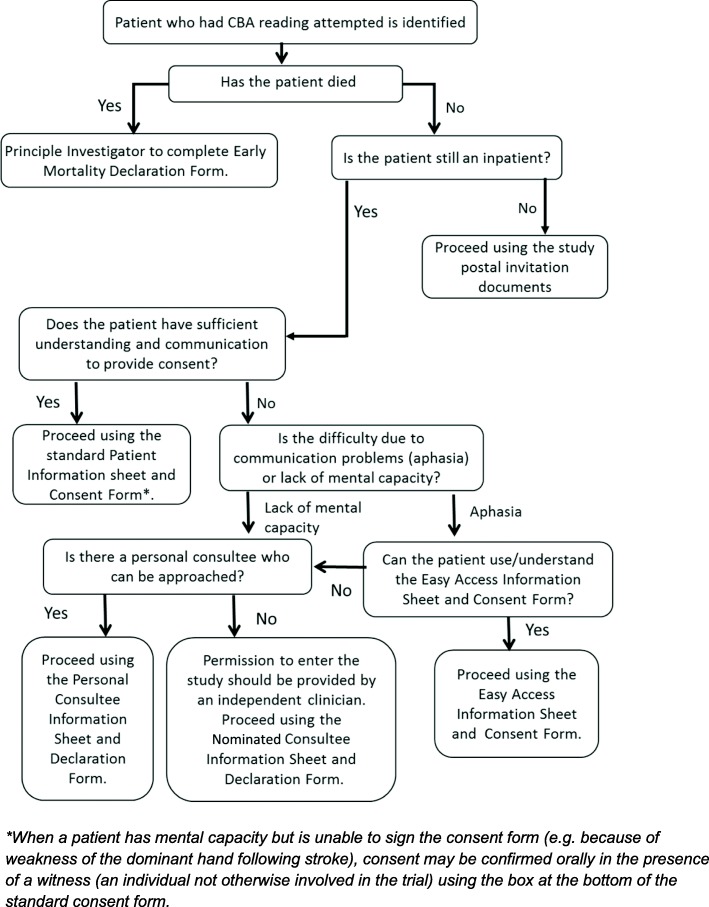
Decision process for study consent

Figure 1 summarises the decision process for obtaining study consent.

### Cerebral Bioimpedance Asymmetry measurement process (index test)

The Cerebrotech Visor System™ consists of two major components: a scanner, worn like a pair of eyeglasses over the forehead, and a ‘tablet’ computer, which serves as the system’s controller and display. Within the scanner, two radio emitter antennas are located at the back of the head and a receiver antenna is positioned at the forehead. No electrodes or metal come into direct contact with the patient. Disposable sleeves cover the plastic housing in contact with the patient to prevent skin contamination between users. In addition, a cradle pillow (with disposable sleeves) to be used for keeping the patient’s head centred and comfortable during the reading is part of the CVS.

The scanner consists of electronic modules for generating radiofrequency waveforms, receiving the signals, and performing associated operational functions. It also has a rechargeable battery. The tablet running the monitoring software wirelessly controls the device through a Bluetooth® connection and captures scan data. During operation, the waveform generator supplies radiofrequency signals to the emitter antennas and records the signals acquired by the receiver antenna.

During a measurement, the tablet is turned on and the CerebroScan™ software application is launched. The scanner is switched on and placed on the patient’s head. The CerebroScan interface screen has a click-box to start the scan sequence, which is executed automatically. Raw bioimpedance data are transmitted from the scanner to the tablet. The patient wears the scanner for 2–3 min altogether. The operator subsequently removes the scanner and turns off its power.

In order to create the numerical value for CBA (continuous data values 0–100), raw data from the CVS must be further analysed by software held by the device manufacturer (Cerebrotech Medical Systems Inc). Raw data will be transmitted by the tablet to the manufacturer via the internet. These data are not identifiable as each patient’s reading will be labelled with a unique study ID only. Prior to transfer of data, CVS software asks staff to confirm that patient consent is in place. Only data where this is confirmed will be transmitted. Data for non-consented patients will be deleted at this point and not transferred. The manufacturer will return the CBA reading to the researchers at Newcastle University Stroke Research Group who are leading the project. At no point will the device reading be made available to the hospital clinical team such that it could influence patient care. The device reading will similarly not be available to clinicians/researchers performing the brain imaging review or attribution of reference standard outcome states (see ‘Reference standard test (comparator)’ section below) until these data have been finalised. No clinical data will be available to the manufacturer prior to provision of a CBA reading.

### Reference standards

Reference standard tests are required for key outcome states which constitute the suspected stroke population: large volume intracerebral haemorrhage (LICH), small/medium intracerebral haemorrhage, ischaemic stroke with LVO (LVO), ischaemic stroke without LVO, symptomatic severe anterior vessel stenosis (SSAVS), transient ischaemic attack (TIA), mimic conditions and prior territorial stroke (PTS). In clinical practice, some of the acute outcome states can overlap (e.g ischaemic stroke or TIA can be caused by SSAVS), and all acute outcome states may co-exist with a prior territorial stroke. As only one outcome state can be allocated for study analyses, non-overlapping discrete definitions and reference standards have been assigned for each acute outcome state and an attribution process developed to describe when PTS will be the state assigned. The definitions and attribution process result in the presence of any ‘complex stroke’ (i.e. ischaemic stroke with LVO or SSAVS or LICH ≥ 60 ml in volume or PTS) being dominant over other states which are in keeping with the intended purpose of the CVS test.

Whilst brain imaging tests are available which objectively confirm any intracerebral haemorrhage (CT), LVO (CT/MR angiography), and PTS (CT), no single diagnostic test exists for ischaemic stroke without LVO, SSAVS, TIA and mimic conditions. For SSAVS, the reference standard will be based on brain imaging plus clinical report of the side of the body affected by focal neurological symptoms. For ischaemic stroke without LVO, TIA and mimic conditions, the reference standards will be based on expert clinician diagnosis informed by brain imaging +/− other investigations as clinically appropriate. Full details are given below and a summary of the reference standards to be used is shown in Table 1.

**Table 1:** Summary of clinical outcome states and their reference standards

	Outcome state	Reference standard
Complex	Ischaemic stroke with large vessel occlusion	CT/MR angiography assessed by blinded neuro-radiologist. LVO defined as a Ten Point Clot Burden Score <10.
	Large volume intracerebral haemorrhage	Brain imaging assessed by blinded neuro-radiologist. Haematoma volume of at least 60ml in a single cerebral hemisphere.
	Prior territorial stroke	Brain imaging assessed by blinded neuro-radiologist. Appropriate changes in either cerebral hemisphere.
	Symptomatic severe anterior vessel stenosis	CT/MR angiography assessed by blinded neuro-radiologist which shows ≥ 70% narrowing of any large branch of the anterior cerebral circulation (ICA or MCA) without occluding thrombus, the side of the focal neurological symptoms suffered by the patient is compatible with stenosis, the severity of focal symptoms is ≥ 6 on NIHSS.
Minor Stroke	Small / medium volume intracerebral haemorrhage	Brain imaging assessed by blinded neuro-radiologist. Haematoma volume < 60ml in a single cerebral hemisphere.
	Ischaemic stroke without large vessel occlusion	Expert clinician diagnosis at 72 hours after hospital admission (or discharge/death if sooner), CTA/MRA confirms the absence of LVO (i.e. Clot Burden Score = 10), and CTA/MRA plus clinical findings confirm the absence of SSAVS.
Non Stroke	Transient ischaemic attack	Expert clinician diagnosis at 72 hours after hospital admission (or discharge/death if sooner) and brain imaging findings do not refute the clinician opinion or indicate the presence of one of the other acute outcomes.
	Mimic condition	Expert clinician diagnosis at 72 hours after hospital admission (or discharge/death if sooner) and brain imaging findings do not refute the clinician opinion or indicate the presence of one of the other acute outcomes.

#### Neuro-imaging defined outcome states

It is intended that all participants in this study will undergo at least one brain-imaging test. According to clinical indications, some participants will undergo further imaging investigations. All imaging performed within 72 h of admission will undergo blinded expert review by an experienced neuro-radiologist. Where imaging alone is the reference standard test to determine the presence of an outcome, the blinded review report(s) will be used. Although local routine imaging reports will also be obtained, these will not alter the data to be used in the main study analyses. The frequency and nature of any discrepancies between the reports will, however, be reported on study completion.

Ischaemic stroke due to LVO will be defined as present if CT or MR angiography has been conducted and demonstrates reduced filling in any large branch of the anterior cerebral circulation. This will be assessed and recorded by a neuro-radiologist blinded to patient and study information using the Ten Point Clot Burden Score [24]. A score < 10 will indicate presence of LVO. Angiography is not routinely performed for all suspected stroke patients as clinical or other radiological examination can preclude the need for the investigation. It is routinely performed for participants where plain CT has not shown a haemorrhage or another radiological diagnosis for the acute symptoms, e.g. tumour.

Intracerebral haemorrhage will be defined as present if a small (approximately < 30 ml), medium (approximately 30 to < 60 ml) or large (approximately ≥ 60 ml) volume haematoma is recorded during CT (+/− MRI) review by a neuro-radiologist blinded to patient and study information. The haematoma volume will be estimated using the standard ABC/2 formula where A is the greatest haemorrhage diameter by CT, B is the diameter 90° to A, and C is the number of CT slices with haemorrhage multiplied by the slice thickness (with approximate allowance for any inter-slice gap) [25].

Prior territorial stroke will be defined as present if an appropriate non-lacunar lesion of any size exists on brain imaging in either cerebral hemisphere (i.e. chronic hemispheric volume loss reflecting previous non-lacunar infarction or ICH). If PTS is present, the approximate proportion of volume loss in each hemisphere will be recorded (< 24%; 25–49%; 50–74%; > 74%).

Blinded review of images will also record the presence of additional relevant features, e.g. interventricular haemorrhage, subarachnoid haemorrhage, occlusion of large posterior vessels (e.g. vertebral, basilar), cerebral/cerebellar volume loss, but these features will not be used to define outcome states.

#### SSAVS outcome state

SSAVS will be defined as present if (a) CT or MR angiography has been conducted and this demonstrates that there is ≥ 70% narrowing of any large branch of the anterior cerebral circulation (internal carotid artery or middle cerebral artery) without occluding thrombus, (b) the side of the focal neurological symptoms suffered by the patient are in keeping with the imaging (e.g. the stenosis is present in the circulation on the left and the symptoms were on the right), and (c) the severity of the focal symptoms is ≥ 6 on the NIHSS scale (measured on admission).

#### Expert clinician diagnosis defined outcome states

As no single test exists for ischaemic stroke without LVO, transient ischaemic attack (TIA) and mimic conditions, expert clinician opinion informed by brain imaging +/− other investigations as appropriate will be used as the reference standard.

An expert clinician diagnosis will be recorded for all participants in this study although (as described above), this will not be used to define the presence of LVO, intracerebral haemorrhage, PTS or SSAVS outcomes.

Because primary medical diagnoses recorded in medical records can vary according to the taxonomy used and the terminology preferred by individual clinicians, e.g. chest infection is synonymous with pneumonia, bronchopneumonia and lower respiratory tract infection, clinicians will be asked to select a ‘definite’ or ‘probable’ primary clinical diagnosis according to a pre-defined framework as shown in Table 2. As diagnoses are sometimes uncertain for a time after admission to the hospital, clinicians will be asked to provide a diagnosis assigned at 72 h after hospital admission (or discharge/death if sooner). The framework will also include an option for ‘unclear’ if the clinician cannot assign a diagnosis. If ‘unclear’ is selected, adjudication by two further stroke team clinicians will be requested to attempt resolution. If this is not possible, ‘unclear’ will still be recorded.

**Table 2:**
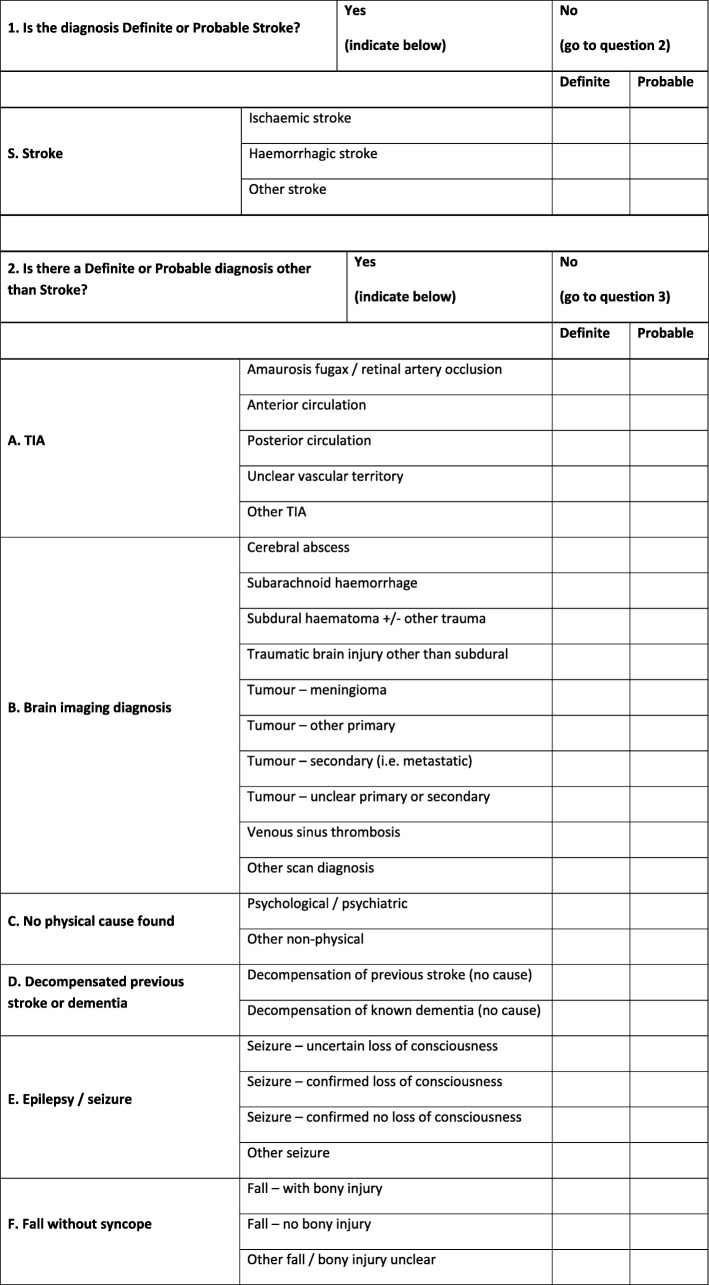

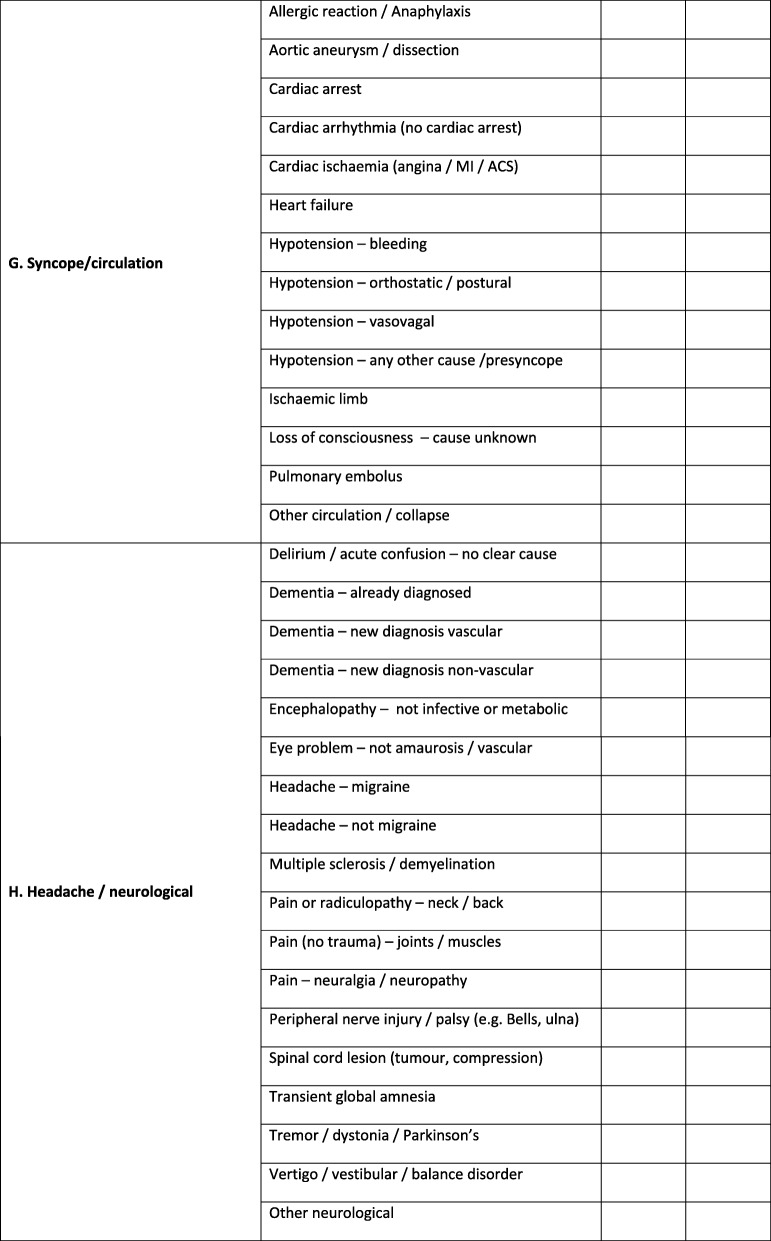

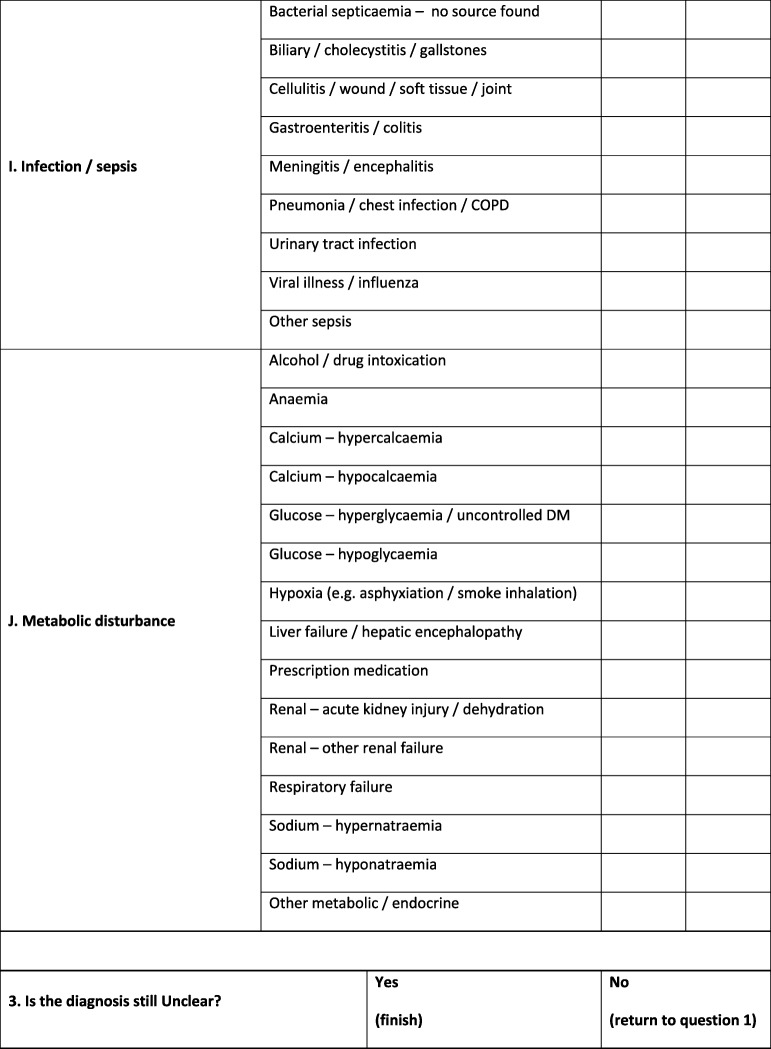
Pre-defined primary diagnosis framework

Ischaemic stroke without LVO will be defined as present if the expert clinician considers the patient to have a ‘definite’ or ‘probable’ ischaemic stroke, CT/MR angiography confirms the absence of LVO (i.e. clot burden score = 10), and CT/MR angiography plus clinical findings confirm the absence of SSAVS.

It is possible that there will be participants with a clinical diagnosis of ischaemic stroke who do not undergo CT/MR angiography for some reason (e.g. due to agitation). In these cases, the LVO and SSAVS status are unknown. Data from these participants will not be included in any analysis of operating characteristics but their clinical and radiological information and CBA data will still be reported.

A transient ischaemic attack will be defined as present if the clinician records any ‘definite’ or ‘probable’ TIA diagnosis using the pre-defined framework and brain imaging does not contain findings that refute this opinion or indicate the presence of one of the other acute outcomes. If there is any disagreement between the blinded neuro-radiological review of imaging performed and a clinical diagnosis of TIA, the imaging diagnosis will be used to determine the outcome but such discrepancies will also be reported.

A stroke mimic condition will be defined as present if the clinician records any ‘definite’ or ‘probable’ non-stroke/non-TIA diagnosis using the pre-defined framework and brain imaging is compatible (e.g. tumour) plus does not contain findings which indicate the presence of one of the other acute outcomes. If there is any disagreement between the blinded neuro-radiological review of imaging performed and a clinical diagnosis of mimic condition, the imaging diagnosis will be used to determine the outcome but such discrepancies will also be reported.

If a clinician records an ‘unclear’ diagnosis for any patient and this cannot be resolved, these participants will not be included in any analyses of operating characteristics as it has not been possible to determine an outcome. Available data for these participants will still be reported.

#### Outcome state attribution process

**Fig. 2:**
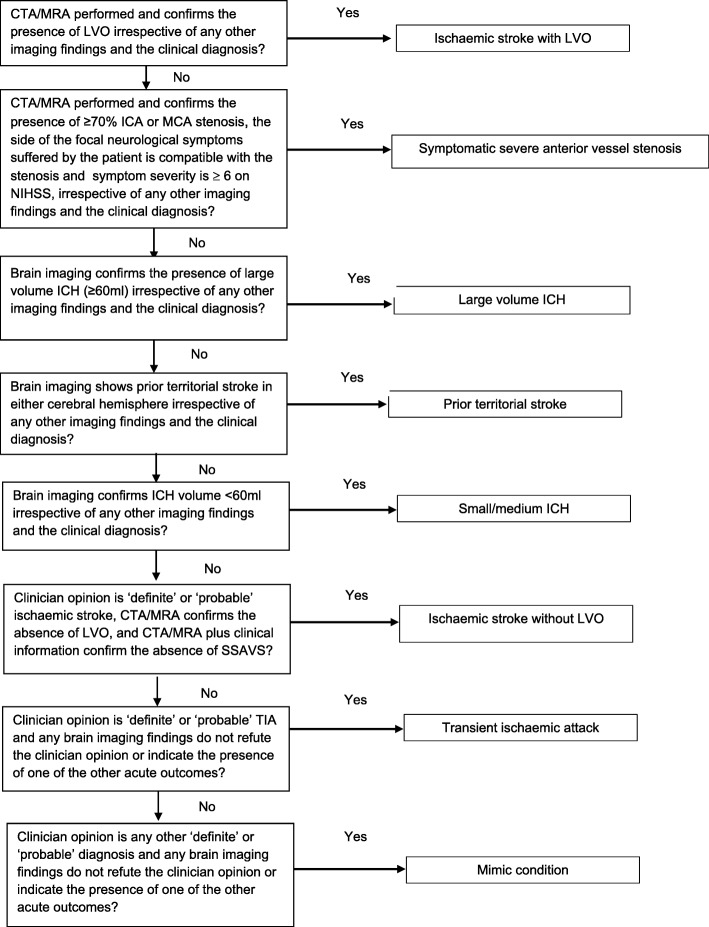
Decision tree for assigning the outcome state to be used in the main study analyses

Participants with a clinical diagnosis of ischaemic stroke who have not had a CTA/MRA conducted will not have an outcome state attributed because their LVO or SSAVS status cannot be established.  Where participants have evidence (as described by the reference standards above) of a new LVO, LICH or SSAVS, these will be the outcome states assigned. For other participants, if PTS is present irrespective of other outcome states, PTS will be assigned. Where participants do not have PTS, they will be assigned small/medium intracerebral haemorrhage, ischaemic stroke without LVO, TIA or mimic condition according to the reference standard criteria described above. The attribution process is illustrated as a decision tree in Fig. 2.

### Study data collection

For patients who give consent for enrolment in the study, CBA measurement data, imaging data and routine healthcare data to confirm study eligibility and conduct study analyses will be collected. CBA measurement data will be captured by the CVS device and subsequently harvested to be linked with clinical data. The CBA measurement process, imaging data and other routine healthcare will be recorded onto study-specific case record forms by hospital staff trained to deliver this research project.

Data from case record forms will be entered locally onto a secure online database maintained by Newcastle University Stroke Research Group. Participants will be identified by a unique study number only in both the paper case record form and study database. Where consent is not obtained, case record form data recorded about the CBA measurement process (which took place prior to consent) will be destroyed and not uploaded onto the online database. As detailed in ‘Cerebral Bioimpedance Asymmetry measurement process (index test)’ section above, data from the CVS device will be deleted.

#### Data about the CBA measurement process

Date/time of attempted CBA measurement; location of CBA measurement attempt (accident/emergency department/medical admissions unit/acute stroke unit/CT scan room/other); patient head position during CBA reading (CBA cradle used/hospital pillow used/both cradle and hospital pillow used/no head support used/other); angle of the head of the bed during CBA measurement (15 °/30 °/45 °/60 °/unknown); CBA data obtained (yes/no); if data was obtained, any pertinent events which took place during the reading (e.g. sneezing, coughing, head tilting); if data were not obtained, reason why abandoned (patient agitation/insufficient time before brain imaging/change in condition of the patient/scanner or tablet malfunction/other); complications from CBA measurement (yes: skin irritation; injury; corneal abrasion; other free text/no)

#### Data related to the first 24 h of admission

Data for confirmation of inclusion and exclusion criteria:

Demographic information (age); date/time of hospital attendance; confirmation of admission by ambulance (yes/no); confirmation that ambulance personnel suspected stroke (yes/no); symptom onset or last known to be well date/time (hospital judgement); first recorded conscious level on arrival at hospital (AVPU scale); first blood glucose reading on admission (capillary or serum glucose); confirmation of other inclusion and exclusion criteria (e.g. brain imaging will be performed, absence of electro-stimulating devices in the head and neck).

Other data related to day 1 of admission:

Demographic information (gender); symptoms suggesting possible stroke according to the ambulance clinician (facial weakness; arm weakness; speech disturbance; other); side of symptoms according to the ambulance clinician (left/right/both/not applicable, e.g. speech); first blood pressure reading on admission; first heart rate reading on admission; first body temperature reading on admission; first peripheral oxygen saturation on admission; ECG rhythm (presence of atrial fibrillation); stroke symptom severity as soon as possible after arrival (National Institute of Health Stroke Score (NIHSS) including all component scores [7]); time of NIHSS completion; clinical stroke type according to new symptoms (Oxford Community Stroke Project classification including left/right lateralisation as appropriate [26]); previous medical history from healthcare records (stroke; head injury; intracranial surgery; TIA; atrial fibrillation; diabetes; hypertension; hyperlipidaemia); if a previous stroke was recorded, had it resulted in permanent arm or leg weakness (no; yes left; yes right; yes both), or any permanent speech difficulty (yes or no); current use of anticoagulant medication; pre-stroke modified Rankin Score [27]; pre-stroke walking ability (independent; walking aid; physical assistance from one person; unable to walk at all); diagnosis listed after completion of initial assessments (stroke/non-stroke (if non-stroke free text for diagnosis); thrombolysis treatment administered (yes/no); date/time of bolus administration (if thrombolysis received); mechanical thrombectomy treatment administered (yes/no); date/time of arterial puncture (if thrombectomy received).

#### Data related to 72 h after admission (or death/discharge if this is sooner)

Deceased, inpatient or discharged alive at 72 h after admission; if deceased, cause of death according to death certificate; if discharged/deceased, discharge/death date; confirmation that symptom onset time recorded in data related to first 24 h of admission is still correct at 72 h after admission or death/discharge if sooner (yes/no, if no, new date/time); primary medical diagnosis in place at 72 h after admission or death/discharge if sooner, recorded according to the pre-defined framework (see ‘Reference standard test (comparator’ section above); new complication of CBA measurement documented by 72 h after admission or death/discharge if sooner (yes: skin irritation; injury; corneal abrasion; infection; other free text/no); follow-up information about any complication previously noted (free text).

#### Imaging data

Brain imaging performed in the first 72 h of attendance (CT/MR/CTangiograpy /MRangiography/none); brain imaging date(s) and time(s); brain imaging result(s) (formal report/documentation in the medical records if no formal report plus selection of infarction, haemorrhage or other); for participants with a diagnosis of ischaemic stroke at 72 h (or death/discharge if sooner): CT or MR angiography performed on admission (yes/no); for ischaemic stroke without angiography: reason why angiography was not performed (stroke too mild/plain CT imaging changes too advanced/patient clinically unsuitable for thrombectomy: state reason/thrombectomy unavailable in time for treatment/other)

Anonymised brain images will also undergo separate blinded neuro-radiologist review using a standard checklist to record: changes of cerebral ischaemia including Alberta Stroke Program Early CT Score (ASPECTS) score [28]; haemorrhage including approximate haematoma size using the ABC/2 approach [25]; prior territorial stroke in either cerebral hemisphere (occupying < 24%; 25%–49%; 50%–74%; > 74% on each side); other pathological findings (e.g. subdural haematomas, tumours, subarachnoid blood).

If angiography is performed, the following will also be recorded: Ten-Point clot burden score [24]; extended thrombolysis in cerebral infarction scale [29]; absence/presence of anterior vessel stenosis and approximate percent of maximal luminal narrowing (< 50%; 50–69%; 70–99%; 100%); approximate ASPECTS Collateral Score (an approximation because no additional imaging sequences will be done specifically to examine collateral flow) [30].

### Blinding

Patients, clinicians and hospital research staff will be blinded to CBA results. Neuro-radiologists responsible for providing the independent imaging reports and research clinicians responsible for the outcome state attribution process will also be blinded to CBA results. In addition, staff from Cerebrotech Medical Systems Inc who will provide CBA readings from raw measurement data will not have access to any clinical data prior to provision of a CBA reading.

### Staff training and awareness

Study-specific training will be provided for stroke clinicians and hospital research staff. This will cover participant identification, use of the CVS, consent, and completion of the study documentation and database as appropriate.

### Study withdrawal

No specific withdrawal criteria have been pre-set. Participants may withdraw from the study at any time for any reason. Data collected prior to withdrawal will be used in the study analysis unless the patient or their representative requests that this should not be the case. Should a decision to withdraw from the study be made, a reason for withdrawal will be sought but participants can choose to withdraw without providing an explanation.

### Safety evaluation

This is a clinician-blinded observational study of a CE-marked diagnostic technology that has already been used in a clinical setting and will not change patient treatment. The risks from participation should be no greater than standard clinical care. The measurement of CBA takes less than 3 min and occurs in parallel with the clinical assessment process, so will not cause any meaningful delay in the provision of standard care.

There are no known complications following exposure to low-energy electromagnetic fields. During the assessment, there is brief superficial contact between patients and the portable CVS device, which itself does not contain any biological or hazardous materials. Any risk of cross-infection is eliminated by a disposable sleeve covering wherever the frame of the scanner is in contact with the patient’s skin. No safety issues were reported during proof of concept studies in North American hospital and ambulatory settings (*n* = 235), which specifically sought evidence of skin irritation, injury, infection and corneal abrasion [21].

Study data collection will include documentation of any complications following the immediate use of the device. Follow-up information about any such complications will be sought at 72 h after admission. Additionally, routine data collection will include a check for any new complications by 72 h after admission.

Should a medical event occur which is serious (results in death; is life-threatening; requires inpatient hospitalisation or prolongation of existing hospitalisation; results in persistent or significant disability or incapacity; consists of a congenital anomaly or birth defect; otherwise considered significant by investigator) and is perceived to be related to the use of the CVS, a separate study Serious Adverse Event form will be completed. All such events will be considered ‘unexpected’ and reported to the chief investigator, study sponsor and Research Ethics Committee.

### Statistical analysis

1. Objective 1: To determine the diagnostic accuracy of CBA measurement performed using the CVS to identify complex stroke (ischaemic stroke with LVO or SSAVS or LICH ≥ 60 ml in volume or PTS) in patients arriving at the hospital with a paramedic assigned diagnosis of suspected acute stroke.

In the recently published work using this technology, a threshold of CBA = 10 was derived as optimal for identification of complex stroke [21]. This threshold will be validated on the data collected in this study and considered acceptable if it results in specificity of at least 80% and sensitivity of at least 70%. These values have been chosen as clinically important and stringent, to allow validation to be conducted. Sensitivity and specificity will be reported with 95% confidence intervals.

However, the population to be recruited in this study may differ from the earlier work (e.g. less time has elapsed since stroke onset). Therefore, the threshold of CBA = 10 may not result in meeting the criteria described above. If this is the case, a new threshold will be estimated. Univariate logistic regression analysis with diagnosis (complex stroke) as the outcome (*y*) and CBA measurement as the explanatory variable (*x*) will be used to construct an empirical receiver operating characteristic (ROC) curve for all possible thresholds. A new threshold will be chosen to maximize specificity while aiming to maintain a clinically acceptable level of sensitivity (70% in this instance). Sensitivity, specificity and ROC area under curve (AUC) (with confidence intervals) will be reported for the chosen threshold.

2. Objective 2: To determine the diagnostic accuracy of CBA measurement performed using the CVS to identify LVO in patients arriving at the hospital with a paramedic assigned diagnosis of suspected acute stroke.

For this objective, analyses as described above for objective 1 will be conducted, i.e. a threshold of CBA = 10 will initially be evaluated but a new threshold may be estimated if CBA = 10 does not result in sensitivity and specificity meeting the stated criteria.

3. Objective 3: To explore key clinical and radiological influences upon the diagnostic accuracy of CBA measurement for complex stroke and LVO.

This is an exploratory objective, and as such the analyses will be data-driven and dependent on results for objectives 1 and 2. Proposed investigations focus on three main areas: (i) whether available clinical data might be incorporated to improve the diagnostic accuracy of the CVS; (ii) variations in specification of the reference diagnosis for LVO, and (iii) patient subgroup analyses to identify whether the CVS may perform better in some clinical settings than others.

(i) Clinical covariates: key variables will be added to the logistic regression models described in objectives 1 and 2 to see if this could significantly increase the accuracy of the prediction of the model. Clinical variables that would be available to ambulance personnel or staff involved in urgent assessment at the hospital will be included to reflect those available at the point of testing. Multivariate logistic regression will be conducted, with diagnosis (complex stroke OR LVO) as the outcome (*y*) and CBA measurement along with variables considered to be of possible clinical importance (such as gender, age, NIHSS) as the explanatory variables (*x*1, *x*2, *x*3, …). A stepwise variable selection procedure will be used, and only variables that significantly improve the ROC AUC will be retained in the model. Sensitivity, specificity, ROC AUC (with confidence intervals) and threshold will be reported if a suitable model is found.
(ii) Alternative definitions for LVO: the diagnostic accuracy of CBA measurement will be assessed by modelling as described for objective 1, where the reference diagnosis will be assigned as follows:
  Radiological definition: At the time of the data analysis, if the clinical consensus regarding size and distribution of clot that is feasible for thrombectomy (described using the clot burden score and/or the extended TICI scale) has changed, the alternative definition(s) will be used to re-categorise participants as LVO present or absent.
  Best clinical standard for LVO identification: currently, the most accurate clinical scale for the indication of the presence of LVO is the Rapid Arterial oCclusion Evaluation (RACE) [31]. A numerical RACE score will be calculated retrospectively using the hospital NIHSS (which contains all score items), as RACE is not routinely in use in the UK and will not be used in this study. RACE scores will be used to re-categorise participants as LVO present or absent based upon a published cut-off value.
(iii) Exploratory subgroup analysis: if data permit, this will be carried out by graphical presentation of the CBA results. The proposed subgroups to investigate are:

- Acute stroke: patients with a diagnosis of acute stroke will be evaluated (i.e. after exclusion of patients with PTS, TIA and mimic conditions).
- Acute IS: patients with acute ischaemic stroke will be evaluated (i.e. after exclusion of patients with TIA, mimic conditions, PTS and haemorrhage).
- Exclusion of participants having posterior circulation stroke: participants with posterior circulation stroke, defined by the Oxford Community Stroke Project (OCSP) classification and verified against relevant NIHSS components will be excluded. Clinically, these individuals would be considered unlikely to have anterior circulation vessel occlusion or haemorrhage, and might not have a CBA reading performed as part of their emergency assessment. The remaining population will be evaluated.

Further exploratory analyses may be undertaken but these analyses will be data-driven and reported as such.

4. Objective 4: To report the technical failure rate of the CVS.

Technical and clinical reasons will be reported for why a CBA reading was attempted but not obtained. Technical reasons will include device failure resulting in no data acquisition, and collection of erroneous raw data leading to calculation of a CBA value which is perceived to have an implausible bioimpedance pattern.

Data from the following groups will be reported but excluded from the analyses:

- Participants with ischaemic stroke but without CT angiography or MR angiography, as their LVO state is unknown.
- Participants with an expert clinician diagnosis of ‘unclear’ which cannot be resolved as it has not been possible to assign a diagnosis.
- Following attendance at the hospital, other information sources may reveal retrospectively that one or more of the study eligibility criteria have not been met (e.g. the symptom onset was more than 6 h ago). This is not uncommon during emergency care assessments due to the accuracy of information immediately available. Participants who do not meet the inclusion criteria according to information available by 72 h after admission will not contribute towards the analyses.
- Participants where a CBA reading is attempted but this is unsuccessful (the CVS alerts the operator to failure to capture data).
- All CBA readings obtained will be checked for a plausible bioimpedance pattern. Participants with CBA readings who are perceived to be implausible will not be included in the analyses, e.g. readings obtained from patients with previously unknown cranial metallic implants.

#### Sample size

The future intended purpose of CBA measurement is to ‘rule in’ patients with complex stroke and/or LVO from the suspected stroke population. The sample size calculation is therefore based on the detection of test specificity. ‘Rule in’ is chosen as the test results would be used to redirect individuals to a neuroscience centre for highly specialist care. Suspected stroke patients without a diagnosis of complex stroke or LVO do not need transportation to a neuroscience centre and should be admitted to the nearest local hospital.

Reported frequencies for LVO, large intracerebral haemorrhages and prior territorial stroke are 27%, 3% and 4% respectively amongst the unselected population with suspected stroke. There are no reliable estimates for the frequency of SSAVS in the unselected UK population—it is an uncommon finding and previous cohorts of LVO patients are likely to have included cases with high degrees of symptomatic stenosis without reporting them separately. Therefore, using the above-reported frequencies and a minimum specificity of 70% from the diagnostic test, alpha = 0.05 and beta = 0.1:

- One hundred twelve participants would detect a specificity of 85% (95%CI: 70%–100%) for complex stroke (i.e. LVO + SSAVS + LICH + PTS) versus all other outcomes in the suspected stroke population (i.e. small/medium intracerebral haemorrhage + ischaemic stroke without LVO + TIA + mimic condition).

- One hundred one participants would detect a specificity of 85% (95%CI: 70%–100%) for ischaemic stroke due to LVO versus all other outcomes in the suspected stroke population (i.e. SSAVS + LICH + PTS + small/medium intracerebral haemorrhage + ischaemic stroke without LVO + TIA + mimic condition).

In terms of sensitivity, the 112 participants would detect sensitivity of 85% (95%CI: 63%–100%) for complex stroke and 85% (95%CI: 61%–100%) for LVO.

As outlined above, some enrolled participants may not provide data for inclusion in the analyses and this lack of data is difficult to estimate. The initial recruitment target is inflated for 10% data exclusion (124 participants) but prospective monitoring of the number of cases which can contribute to the analyses will occur and fewer or additional participants will be sought if required.

### Study monitoring, quality control and quality assurance

The Chief Investigator will have overall responsibility for study conduct. The local Principal Investigator will be responsible for the day-to-day study conduct at the NHS site. The study will be managed by a coordinating centre based at Newcastle University Stroke Research Group who will provide training and day-to-day support for the NHS site. Quality control will be maintained through adherence to Newcastle Biomedicine Clinical Research Platform SOPs, the study protocol and research governance regulations. The study may be subject to inspection and audit by Newcastle upon Tyne Hospitals NHS Foundation Trust under their remit as sponsor.

### Dissemination of results

The study will be presented at national and international conferences and reported in peer-reviewed journals. Reports will be written for the study funder, sponsor and regulatory bodies. A lay summary of the results will be available for study participants.

## Discussion

There is substantial high-quality evidence that early access to thrombolysis, thrombectomy and specialist care improve stroke outcomes. However, stratification of patients with suspected stroke is challenging and this can lead to delays to care. Cerebral Bioimpedance Asymmetry measurement using the Cerebrotech Visor™ System may enable rapid identification of certain stroke types that benefit from time-critical treatment. In particular, a quick and portable test for LVO could significantly improve access to thrombectomy with subsequent improved outcome improved outcome from this stroke type. This study will provide data about the diagnostic accuracy of CBA measurement for LVO and other types of complex stroke.

### Study status

At the time of submission of this manuscript, the study is in setup. Recruitment is scheduled to commence in October 2019. Protocol version 3 dated 11th May 2019 was used to prepare this manuscript.

## Data Availability

Not applicable.

## Abbreviations

ASPECTS: Alberta Stroke Program Early CT Score AUC Area under curve AVPU Alert Voice Pain Unresponsive CBA Cerebral Bioimpedance Asymmetry CT Computerised tomography CVS Cerebrotech Visor™ System HASU Hyperacute stroke unit ISRCTN International Standard Randomised Controlled Trials Number LICH Large volume intracerebral haemorrhage LVO Large vessel occlusion MRI Magnetic resonance imaging NHS National Health Service NIHSS National Institute of Health Stroke Scale PTS Prior territorial stroke ROC Receiver Operating Characteristic SSAVS Symptomatic severe anterior vessel stenosis STARD Standards for the Reporting of Diagnostic accuracy studies TIA Transient ischaemic attack
